# Conservation implications for dingoes from the maternal and paternal genome: Multiple populations, dog introgression, and demography

**DOI:** 10.1002/ece3.3487

**Published:** 2017-10-19

**Authors:** Kylie M. Cairns, Sarah K. Brown, Benjamin N. Sacks, J. William O. Ballard

**Affiliations:** ^1^ School of Biotechnology and Biomolecular Sciences University of New South Wales Sydney NSW Australia; ^2^ Mammalian Ecology and Conservation Unit Veterinary Genetics Laboratory School of Veterinary Medicine University of California Davis CA USA; ^3^ Department of Population, Health and Reproduction School of Veterinary Medicine University of California Davis CA USA

**Keywords:** Australia, biogeography, conservation, demography, dingoes, hybridization, mitochondrial DNA, mtDNA, population expansion, Y chromosome

## Abstract

It is increasingly common for apex predators to face a multitude of complex conservation issues. In Australia, dingoes are the mainland apex predator and play an important role in ecological functioning. Currently, however, they are threatened by hybridization with modern domestic dogs in the wild. As a consequence, we explore how increasing our understanding of the evolutionary history of dingoes can inform management and conservation decisions. Previous research on whole mitochondrial genome and nuclear data from five geographical populations showed evidence of two distinct lineages of dingo. Here, we present data from a broader survey of dingoes around Australia using both mitochondrial and Y chromosome markers and investigate the timing of demographic expansions. Biogeographic data corroborate the presence of at least two geographically subdivided genetic populations, southeastern and northwestern. Demographic modeling suggests that dingoes have undergone population expansion in the last 5,000 years. It is not clear whether this stems from expansion into vacant niches after the extinction of thylacines on the mainland or indicates the arrival date of dingoes. Male dispersal is much more common than female, evidenced by more diffuse Y haplogroup distributions. There is also evidence of likely historical male biased introgression from domestic dogs into dingoes, predominately within southeastern Australia. These findings have critical practical implications for the management and conservation of dingoes in Australia; particularly a focus must be placed upon the threatened southeastern dingo population.

## 1 INTRODUCTION

The effect of removing large socially complex apex consumers such as whales, big cats, bears, wolves, and dingoes from ecosystems is poorly documented (Estes et al., 2011). Apex predators are in decline, globally, which has lead to and threatens continuing impacts to entire ecosystems (Estes et al., 2011; Morris & Letnic, 2017; Ripple et al., 2014, 2016, 2017). Estes et al. (2011) suggest that worldwide large apex consumer declines can cause extensive trophic cascading, exacerbated by agricultural land management, widespread habitat degradation, pollution, and ultimately climate change. On the Australian continent, indigenous apex predators went extinct thousands of years ago, leaving the dingo as the sole remaining apex predator on the mainland. As such, the dingo plays a central ecological role. Today, dingoes are threatened by extensive lethal control programs, habitat fragmentation, and genetic dilution from hybridization with domestic dogs (Stephens, Wilton, Fleming, & Berry, 2015).

**Figure 1:**
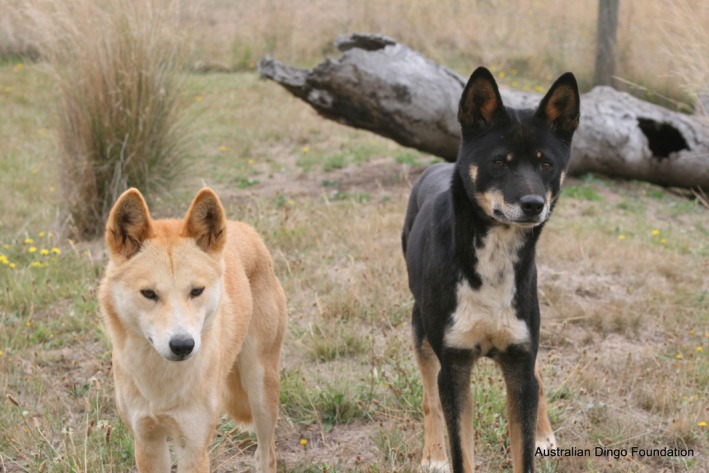
A pair of typical dingoes, as found across the Australian mainland. Dingoes are commonly observed with ginger, white, sable, and black and tan pelage. © Photograph: L. Watson, Australian Dingo Foundation (2017)

In this study, we explore the evolutionary history of Australian dingoes with a goal of informing management and conservation decisions (Figure 1). Since 1788, dingoes have been subject to hybridization pressure from modern domestic dogs brought by Europeans, particularly in regions where human populations are high (Stephens et al., 2015). The observation of hybridization in species and populations is an increasingly common conservation concern; well documented examples include bison, coyotes, wolves, wild cats, and even Galapagos tortoises (Garrick et al., 2012; Halbert & Derr, 2007; Hertwig et al., 2009; vonHoldt et al., 2016; Reich, Wayne, & Goldstein, 1999).

Historically, the dingo is thought to have arrived on mainland Australia approximately 5,000 years before present (BP) (Gollan, 1984; Macintosh, 1975; Savolainen, Leitner, Wilton, Matisoo‐Smith, & Lundeberg, 2004). The minimum arrival time of dingoes is approximately 3,500 years BP based upon the oldest fossil observed in southern Western Australia (Macintosh, 1964). The dingo is frequently blamed for the thylacine extinction on mainland Australia 2,000–3,000 years BP (Fillios, Crowther, & Letnic, 2012; Johnson & Wroe, 2003; Letnic, Fillios, & Crowther, 2012), although new modeling suggests climate change and human population growth may have played a more significant role (Prowse, Johnson, Bradshaw, & Brook, 2014). Dingoes and New Guinea Singing Dog (NGSD) likely came to Oceania with humans. However, there is uncertainty concerning which human colonization event they accompanied and whether dingoes were brought directly to Australia or immigrated via the prehistoric land bridge between Papua New Guinea and Australia prior to 8,000 years BP. It is commonly presumed that dingoes were brought to Australia 5,000 years ago as part of the Neolithic human expansion (Sacks et al., 2013; Savolainen et al., 2004). However, Fillios and Taçon (2016) argue that the Neolithic people are unlikely to be responsible for the arrival of the dingo because Australia lacks key Neolithic cultural indicators.

An alternative hypothesis is that dingoes and NGSD are part of an older dog radiation that immigrated into Australia via the land bridge between Australia and Papua New Guinea, which flooded 6,000–8,000 years BP (Cairns & Wilton, 2016). Some ethnographic evidence supports this hypothesis, for example, the lack of Neolithic cultural items, such as chickens and pigs, in Australia prior to European colonization (Larson et al., 2010; Oskarsson et al., 2011), lack of human genetic signatures indicating contact between South East Asia and Indigenous Australians (Brown, 2013; Haak et al., 2010; van Holst Pellekaan, 2001, 2013; Karafet et al., 2005; McEvoy et al., 2010; Pugach, Delfin, Gunnarsdóttir, Kayser, & Stoneking, 2013), and the finding that dingoes only carry the two ancestral Amylase gene copies, consistent with their having diverged from modern domestic dogs before the agricultural era (Arendt, Cairns, Ballard, Savolainen, & Axelsson, 2016; Freedman et al., 2014). More recent molecular dating efforts based on mitochondrial divergence time suggest that dingoes could have arrived in Australia approximately 8,000–10,000 years BP (Cairns & Wilton, 2016; Oskarsson et al., 2011). Bayesian skyline modeling may help inform this debate by testing when dingoes underwent population expansion and/or contraction.

Knowledge concerning the population biology of dingoes can provide insight into the arrival patterns and origin of this enigmatic canine, which may have important conservation implications. In 2016, Cairns and Wilton identified the presence of at least two dingo lineages, southeastern (SE) and northwestern (NW), in Australia with a pattern of geographical subdivision. Early studies using mitochondrial DNA were unable to elucidate continent‐wide biogeographic patterns, as they restricted genetic sampling to the mitochondrial control region (Oskarsson et al., 2011; Savolainen et al., 2004). Y chromosome studies either had samples from mostly Western Australia or mostly eastern Australia, and differences in genetic marker sampling made it difficult to compare datasets (Ardalan et al., 2012; Sacks et al., 2013).

Ecological theory predicts that shifts in the distribution and abundance of apex predators and herbivores may result in sizable changes in ecosystem dynamics (Fretwell, 1987; Hairston, Smith, & Slobodkin, 1960). In Australia, the distribution and abundance of dingoes is influenced by historical biogeography as well as current hybridization with dogs. This study is the first broad biogeographic survey of dingoes utilizing both mitochondrial and Y chromosome markers that aims to investigate broad patterns of biogeography as a fundamental prerequisite for conservation management. Our data shed light on modern and historical migration and dispersal patterns in male and female dingoes on a continental scale. Bayesian demographic modeling of the distinct dingo lineages suggests the populations may have distinct evolutionary histories, which may impact on conservation. The research has practical implications for dingo conservation and management strategies across Australia, particularly concerning hybridization.

## 2 MATERIALS AND METHODS

### 2.1 Canid sampling

**Figure 2:**
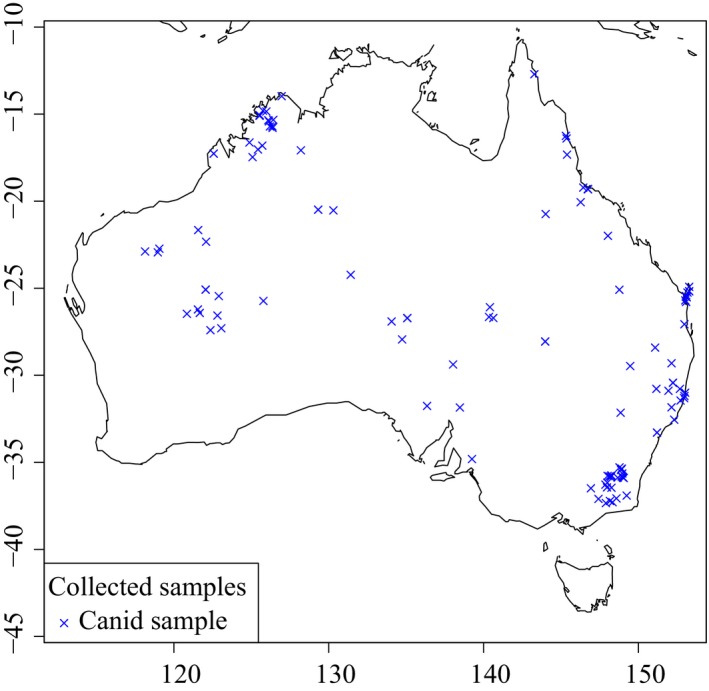
Map depicting geographic sampling of dingoes across Australia. Crosses represent individual samples. New Guinea Singing Dogs are depicted in Papua New Guinea; however, samples were sourced from the North American captive population

In order to investigate biogeography, migration, male and female dispersal patterns, and immigration routes, we sampled 127 dingoes broadly across Australia and five NGSD from the North American captive population (Figure 2, Table 1). Five of the dingoes were sampled from the captive dingo population. We also incorporated a dataset of Y chromosome and mitochondrial control region data from 173 male dogs, including 94 dingoes and 18 NGSD from Sacks et al. (2013).

**Table 1:** Sample data; identifier, geographical locale, latitude, longitude, and genetic identity

Dingo name	Dingo ID	Gender	State	Longitude	Latitude	CR haplotype (collapsed)	CR haplotype (gaps considered)	MtDNA Clade	MtDNA Type	Y chromosome haplotype	GenBank accession #
Alpine 1	96.2	M	VIC	−37.29	148.33	A209	A209	SE	a9	H60‐k11	JX088688
Alpine 2	WD170	M	NSW	−36.46	148.26	A29	A29	SE	a3	H1*/H2*‐k1	JX088692
Alpine 3	44.5	M	ACT	−35.84	148.98	A29	A29	SE	a3	H1*/H2*‐6t	JX088692
Alpine 4	135.10	F	VIC	−37.07	148.58	A29	A179	SE	a12		JX088680
Alpine 5	119.1	M	VIC	−36.17	147.99	A29	A179	NW	d23	H60‐k11	JX088679
Fraser 1	21.3	F	QLD	−25.25	153.17	A29	A179	SE	f1		JX088676
Fraser 2	21.1	M	QLD	−25.17	153.28	A29	A179	SE	f1	H60‐n25	JX088676
Fraser 3	184.4	F	QLD	−25.18	153.28	A29	A179	SE	f1		JX088676
Fraser 4	184.1	M	QLD	−25.7	153.03	A29	A179	SE	f1	H60‐n25	JX088676
Fraser 5	21.4	F	QLD	−25.79	153.08	A29	A179	SE	f1		JX088676
Fraser 6	21.2	F	QLD	−24.915	153.281	A29	A179	SE	a1		KC346413, KC346430
Gibson 1	9.40	F	WA	−26.22	121.55	A203	A203	NW	d5		JX088685
Gibson 2	19.84	F	WA	−27.3	123.06	A29	A29	NW	d5		JX088675
Gibson 3	DE17	M	WA	−26.42	121.67	A200	A200	NW	d5	H60‐n24	JX088673
Gibson 4	DE13	F	WA	−25.45	122.9	A29	A29	NW	d5		JX088687
Gibson 5	18.35	F	WA	−25.08	122.05	A200	A200	NW	d5		JX088672
Kimberley 1	3.45	F	WA	−15.1	125.53	A29	A29	NW	d5		JX088683
Kimberley 2	7.27	M	WA	−16.63	124.88	A9	A9	NW	d7	H3‐n4	JX088691
Kimberley 3	19.11	M	WA	−17.47	125.08	A9	A9	NW	d7	H3‐n20	JX088681
Kimberley 4	4.55	F	WA	−15.35	126.1	din27	din27	NW	d5		JX088682
Kimberley 5	24.96	M	WA	−17.27	122.57	A29	A29	NW	d5	H3‐n4	JX088684
Simpson 1	182.6	F	SA	−26.65	140.35	A29	A179	NW	d5		JX088677
Simpson 2	X1777	M	NT	25.35	133.71	A200	din35	NW	d5	H60‐9k	JX088671
Simpson 3	142.3	F	SA	−27.94	134.74	A200	A200	NW	d4		JX088678
Simpson 4	217.2	F	SA	−26.91	134.06	A200	A200	NW	d14		JX088686
Simpson 5	X1783	F	NT	−24.23	131.42	A200	A200	NW	d5		JX088693
Simpson 6	193.5	M	NT	−26.086056	140.411131	A29	A29	NW	d3	H60‐n25	KC346412, KC346429
Captive 1	Gunyah	M	Captive	—	—	A29	A29	SE	a3	H1*/H2*‐k7	KC346422, KC346439
Captive 2	N858	M	Captive	—	—	A29	A205	NW	d5	H60‐k8	KC346411, KC346428
Captive 3	N895	M	Captive	—	—	A29	A29	SE	a3	H1*/H2*‐n7	KC346422, KC346439
Captive 4	N834	M	Captive	—	—	A29	A29	SE	a3	H3‐12d	KC346422, KC346439
Captive 5	N887	M	Captive	—	—	A29	A198	NW	d5	H60‐n24	KC346411, KC346428
Central Australia 1	X167	M	QLD	−28.06092	143.9805968	din32	din32	NW	d20	H1*/H2*‐6q	JX090189, JX090195, MF784880
Central Australia 2	X179	M	QLD	−28.06092	143.9805968	din32	din32	NW	d20	H1*/H2*‐6q	JX090189, JX090195, MF784880
Central Australia 3	182.2	M	NT	−26.714283	140.627761	A29	A179	NW	d5	H1*/H2*‐6q	KC346411, KC346428
Central Australia 4	197.1	M	SA	−31.854931	138.474641	din32	din32	NW	d5	H1*/H2*‐6q	KC346411, KC346428, MF784880
Central Australia 5	193.2	F	NT	−26.718428	135.073047	A29	A29	NW	d1		KC346416, KC346433
Central Australia 6	193.3	F	NT	−26.712483	135.076839	—	—	NW	d2		KC346424, KC346441
Central Australia 7	TA101	M	NT	−20.49	129.319	A200	A200	NW	d9	H60‐0i	MF774083, MF774092
Central Australia 8	TA91	M	NT	−20.5335	130.29875	A200	A200	NW	d9	H60‐n24	MF774083, MF774092
Central Australia 9	TA94	M	NT	−20.49	129.319	A29	A29	NW	d5	H60‐n25	KC346411, KC346428
Central Australia 10	TA95	M	NT	−20.509	129.462	A29	A29	—	—	H60‐n29	
Central Australia 11	X2060	M	NT	−16.791	137.526	A17	A17	—	—	H3‐6z	
Central Australia 12	150.1	M	SA	−34.814542	139.247263	din32	din32	NW	d21	H60‐k10	MF774086, MF774095, MF784880
Central Australia 13	155.5	M	SA	−31.759594	136.350219	din31	din31	NW	d5	H60‐n25	KC346411, KC34642, MF784879
Central Australia 14	185.1	M	SA	−29.38	138.03	A29	A179	NW	d22	H1*/H2*‐6q	MF774087, MF774096
Dubbo	146.1r	M	NSW	−32.151371	148.847676	A29	A199	NW	d8	H3‐k9	MF774090, MF774099
Inglewood	X2654	F	QLD	−28.41	151.08	—	—	NW	d16		JX094432, JX094433
Moree	X920	M	NSW	−29.4735	149.4685	A29	A208	SE	a10	H1*/H2*‐6t	JX090188, JX090194
Northeastern 1	WD333	F	QLD	−17.33	145.39	A29	A199	NW	d16		JX094432, JX094433
Northeastern 2	WD386	M	QLD	−12.71	143.28	A29	A207	NW	d16	H60‐n28	JX094432, JX094433
Northeastern 3	WD402	F	QLD	−20.06	146.269062	A201	din34	NW	d16		JX094432, JX094433
Northeastern 4	X2159	M	QLD	−16.2498	145.3214	A29	A207	NW	d17	H60‐n27	JX090184, JX090190
Northeastern 5	X987	F	QLD	−19.3017	146.7258	A29	A179	NW	d16		JX094432, JX094433
Northeastern 6	X980	F	QLD	−19.3117	146.7358	A29	A179	NW	d16		JX094432, JX094433
Northeastern 7	X983	F	QLD	−19.3217	146.7438	A29	A179	NW	d16		JX094432, JX094433
Northeastern 8	X985	M	QLD	−19.2228	146.44217	A29	A179	NW	d16	H60‐n24	JX094432, JX094433
Northeastern 10	157.1	M	QLD	−20.737969	144.007975	A29	A199	NW	d5	H60‐9k	KC346411, KC346428
Northeastern 11	219.2	M	QLD	−16.40125	145.36163	A29	A199	NW	d5	H60‐n27	KC346411, KC346428
Northwestern 1	1.64	M	WA	−26.468389	120.835219	A200	din36	NW	d5	H60‐n17	KC346411, KC346428
Northwestern 2	11.55	M	WA	−27.414	122.36	A29	A29	NW	d15	H60‐n22	KC346415, KC346432
Northwestern 3	3.46	M	WA	−15.061	125.541	A29	A29	NW	d5	H3‐12d	KC346411, KC346428
Northwestern 4	4.53	M	WA	−15.416	126.135	A29	A29	NW	d19	H3‐n21	KC346423, KC346440
Northwestern 5	4.54	F	WA	−15.703	126.362	A29	A29	NW	d10		KC346417, KC346434
Northwestern 6	17.87	M	WA	−16.809	125.712	A202	A202	NW	d6	H3‐n21(?)	KC346425. KC346442
Northwestern 7	21.72	F	WA	−22.33	122.0811	A29	A179	NW	d13		KC346421, KC346438
Northwestern 8	9.39	F	WA	−25.725917	125.780583	A200	A200	NW	d5		KC346411, KC346428
Northwestern 9	24.94	F	WA	−17.273	122.568	A29	A29	NW	d15		KC346415, KC346432
Northwestern 10	18.38	F	WA	−25.08333	122.05	A29	A29	NW	d12		KC346426, KC346443
Northwestern 11	3.47	M	WA	−14.853	125.97	A29	A29	NW	d5	H3‐n4	KC346411, KC346428
Northwestern 12	4.52	F	WA	−15.796	126.372	A29	A29	NW	d5		KC346411, KC346428
Northwestern 13	7.36	F	WA	−15.708944	126.203139	din27	din27	NW	d18		KC346418, KC346435, MF784878
Northwestern 14	21.23	F	WA	−17.0307	125.4269	A29	A29	NW	d5		KC346411, KC346428
Northwestern 15	DE11	F	WA	−26.56755	122.8074	A200	A200	NW	d5		KC346411, KC346428
Northwestern 16	14.95	F	WA	−13.96689	126.95647	A29	A29	NW	d24		KC346419, KC346436
Northwestern 17	3.48	F	WA	−14.798	125.753	A29	A29	NW	d5		KC346411, KC346428
Northwestern 18	4.62	M	WA	−15.333	126.416	A29	A29	NW	d10	H3‐n21	KC346417, KC346434
Northwestern 19	N866	M	WA	−17.076	128.203	A202	A202	NW	d11	H60‐k11	MF774084, MF774093
Northwestern 20	X2581	M	WA	−22.9366	118.9646	A210	din33	NW	d5	H60‐n25	KC346411, KC346428
Northwestern 21	X2583	M	WA	−22.8917	118.141892	A203	A203	NW	d5	H60‐n22	KC346411, KC346428
Northwestern 22	X2601	M	WA	−22.7264	119.06011	A29	A29	NW	d5	H60‐n25	KC346411, KC346428
Northwestern 23	X3291	M	WA	−21.65537	121.57327	A29	A29	NW	d5	H60‐k3	KC346411, KC346428
Southeastern 1	X1267	M	QLD	−21.99	148.03	A213	A213	SE	a10	H1*/H2*‐1c	JX090188, JX090194
Southeastern 2	X1273	F	QLD	27.0667	152.966	A29	A179	SE	a10		JX090188, JX090194
Southeastern 3	X229	F	NSW	−31.4468	152.723	A29	A29	SE	a8		JX090187, JX090193
Southeastern 4	X1020	M	ACT	−35.871771	148.99979	A29	A29	SE	a2	H1*/H2*‐6t	JX090186, JX090192
Southeastern 5	127.1	F	VIC	−37.1	147.417	—	—	SE	a4		KC346420, KC346437
Southeastern 6	96.4	M	VIC	−37.22222	148.16168	A29	A29	SE	a3	H1*/H2*‐k1	KC346422, KC346439
Southeastern 7	184.3	M	QLD	−25.515	153.123333	A29	A179	SE	f2	H60‐n25	KC346410, KC346427
Southeastern 8	144.8	F	NSW	−35.77729	148.24837	A29	A29	SE	a2		JX090186, JX090192
Southeastern 9	144.9	F	NSW	−35.77729	148.24837	A29	A29	SE	a2		JX090186, JX090192
Southeastern 10	X874	F	NSW	−35.745734	148.25977	A29	A29	SE	a2		JX090186, JX090192
Southeastern 11	X931	F	NSW	−35.8547	148.212694	A29	A29	SE	a2		JX090186, JX090192
Southeastern 12	WD192	F	NSW	−35.29261833	148.7790664	A29	A29	SE	a2		JX090186, JX090192
Southeastern 13	X791	F	NSW	−30.42866792	152.2332262	A29	A29	SE	a11		JX090185, JX090191
Southeastern 14	44.2	F	ACT	−35.797513	148.913353	A29	A29	SE	a3		KC346422, KC346439
Southeastern 15	85.1	F	VIC	−37.344511	147.9035156	A29	A29	SE	a3		KC346422, KC346439
Southeastern 16	X296	M	QLD	−25.4779854	153.0553244	A29	A179	SE	f3	H60‐n25	MF774082, MF774091
Southeastern 17	21.5	F	QLD	−25.45	153.067	A29	A179	SE	a1		KC346413, KC346430
Southeastern 18	65.1	F	VIC	−36.27755288	147.8651983	A29	A29	SE	a2		JX090186, JX090192
Southeastern 19	166.4	F	VIC	−36.43853	147.96559	A29	A29	SE	a2		JX090186, JX090192
Southeastern 20	X1006	M	ACT	−35.365882	148.9296677	A29	A29	SE	a3	H1*/H2*‐k1	KC346422, KC346439
Southeastern 21	X1012	M	ACT	−35.885449	149.0373287	A29	A29	SE	a3	H1*/H2*‐6t	KC346422, KC346439
Southeastern 22	X1049	M	ACT	−35.427117	148.8781602	A29	A29	SE	a3	H1*/H2*‐k1	KC346422, KC346439
Southeastern 23	X1050	M	ACT	−35.427108	148.87816	A29	A29	SE	a3	H1*/H2*‐k1	KC346422, KC346439
Southeastern 24	X1062	M	ACT	−35.362466	148.920683	A29	A29	SE	a3	H1*/H2*‐k1	KC346422, KC346439
Southeastern 25	X2279	M	ACT	−35.6406	148.9629667	A29	A29	SE	a3	H1*/H2*‐6t	KC346422, KC346439
Southeastern 26	156.3	M	QLD	−25.090277	148.767356	A29	A29	SE	a7	H60‐9k	MF774088, MF774097
Southeastern 27	W0143	M	NSW	−33.29289321	151.1958311	A29	A29	SE	a3	H1*/H2*‐6t	KC346422, KC346439
Southeastern 28	W0144	M	NSW	−33.29289321	151.1958311	A29	A29	SE	a3	H1*/H2*‐6t	KC346422, KC346439
Southeastern 29	W0151	M	NSW	−36.90873	149.23903	A29	A179	SE	a3	H60‐n24	KC346422, KC346439
Southeastern 30	WD036	M	NSW	−35.777	148.011	A29	A29	SE	a3	H3‐k2	KC346422, KC346439
Southeastern 31	X1311	M	NSW	−31.31306346	152.9537903	A29	A29	SE	a1	H3‐k9	KC346413, KC346430
Southeastern 32	X2256	M	NSW	−35.904304	149.045024	A29	A29	SE	a3	H1*/H2*‐k1	KC346422, KC346439
Southeastern 33	X2389	M	NSW	−31.1867476	152.9664462	A29	A29	SE	a1	H3‐k9	KC346413, KC346430
Southeastern 34	X2405	M	NSW	−30.99843908	153.0199648	A29	A29	SE	a1	H3‐k9	KC346413, KC346430
Southeastern 35	X2482	M	NSW	−31.84246908	152.1376204	A29	A29	SE	a6	H1*/H2*‐6t	MF774089, MF774098
Southeastern 36	X2484	M	NSW	−30.77870485	152.6794424	A29	A29	SE	a1	H60‐n25	KC346413, KC346430
Southeastern 37	X2529	M	NSW	−30.42666371	152.2269422	A29	A29	SE	a1	H1‐k6	KC346413, KC346430
Southeastern 38	X2764	M	NSW	−30.78488	151.15678	A29	A208	SE	a5	H3‐k9	MF774085, MF774094
Southeastern 39	X2792	M	NSW	−32.54665335	152.3076804	A29	A29	SE	a1	H60‐k4	KC346413, KC346430
Southeastern 40	X2931	M	NSW	−30.8945242	151.9407884	A29	A29	SE	a1	H1*/H2*‐6t	KC346413, KC346430
Southeastern 41	X3508	M	NSW	−29.309439	152.142707	A29	A29	SE	a1	H1‐k6	KC346413, KC346430
Southeastern 42	X580	M	NSW	−35.804173	148.1267073	A29	A29	SE	a3	H21*‐7d	KC346422, KC346439
Southeastern 43	X606	M	NSW	−35.88248	148.6595	A29	A29	SE	a3	H60‐k11	KC346422, KC346439
Southeastern 44	16.1	M	VIC	−37.222881	147.531358	A29	A29	—	—	H3‐k2	
Southeastern 45	X2070	M	VIC	−36.486	146.93	A29	A29	SE	a4	H1*/H2*‐6t	KC346420, KC346437
NGSD 1	NGSD4	F	Papua New Guinea	—	—	A79	A79	NGSD	ng1		JX088674
NGSD 2	NGSD2	F	Papua New Guinea	—	—	A79	A79	NGSD	ng1		KC346414, KC346431
NGSD 3	NGSD3	F	Papua New Guinea	—	—	A79	A79	NGSD	ng1		KC346414, KC346431
NGSD 4	NGSD6	M	Papua New Guinea	—	—	A79	A79	NGSD	ng1	H60‐k10	KC346414, KC346431
NGSD 5	NGSD5	M	Papua New Guinea	—	—	A79	A79	NGSD	ng1	H60‐k10	KC346414, KC346431

Blood and/or tissue samples were collected, and all dingoes were screened for genetic purity, using a microsatellite‐based assay for domestic dog introgression (Wilton, 2001; Wilton, Steward, & Zafiris, 1999). Only pure or genetically intact dingoes were allocated to this research project (Stephens, 2011; Wilton, 2001; Wilton et al., 1999).

### 2.2 Mitochondrial gene analysis

Mitochondrial and nuclear phylogenetic analyses found that there are at least two dingo lineages, with eight diagnostic mitochondrial nucleotide differences between them (SE and NW, Cairns & Wilton, 2016). Two mitochondrial DNA regions harboring diagnostic mutations and the mitochondrial control region were amplified and sequenced (Table 2). The two diagnostic regions were selected as they contained three of the eight differences between the SE and NW mitochondrial lineages (Cairns & Wilton, 2016). Nonrandom genetic sampling has the potential to overestimate a posteriori significance so care must be taken in interpreting results. The regions selected were 676 bp (positions 7,685–8,361 including a region of ATP6 and ATP8) and 1,028 bp (positions 14,098–15,126 including a region of cytochrome *b*) in length. The mitochondrial control region is 582 bp (incorporating nucleotide positions 15,458–16,039 as in Savolainen et al. (2004)).

**Table 2:** PCR amplification primers and conditions for mitochondrial PCR amplification and sequencing of the dingo and NGSD

	Primer name	Sequence	Nucleotide position	Reference
Diagnostic region pair 1	G8_F	CCAATGATACTGAAGCTATG	7,340	Designed by KMC
	G8_R	ATTTTAGCAGGTTTGGTTAT	7,915	
Diagnostic region pair 2	G13_R	CTAAAAGTCAGAATAGGCATT	15,150	Designed by KMC
	P16_F	TTCAGAACAATCGCACAACC	13,973	Designed in Wilton Lab^a^
Control region	H15422	CTCTTGCTCCACCATCAGC	15,422	Savolainen et al. (2004)
	L16106	AAACTATATGTCCTGAAACC	16,106	

a Designed by M. Wong during his 2010 Honours Thesis (unpublished data) and supervised by AN Wilton KMC.

Qiagen DNeasy kits (Qiagen Sciences, Germantown, USA) were used to extract DNA, and mitochondrial loci were amplified using PCR (Table 2). Briefly, PCR reactions were carried out in 25 μl containing water, 5× Crimson polymerase buffer (New England Biolabs Inc., MA, USA), 1.5 mmol/L of MgCl_2_, 6.25 pmol of each primer, 7.5 mmol/L of dNTPs, 2.5 U of Taq DNA Polymerase (New England Biolabs Inc., MA, USA), and 20–50 ng of DNA template. All PCR reactions were cycled using the following thermal profile: 98°C for 2 min, 95°C for 3 min (add Taq polymerase), then 95°C for 15 s, 52°C for 1 min, 65°C for 1 min for 10 cycles, then 95°C for 15 s, 52°C for 1 min, and 65°C for 1 min (increase time by 5 s each cycle) for 25 cycles followed by 65°C for 10 min.

Prior to sequencing, PCR amplicons underwent purification by ExoSAP‐IT^®^ (USB Amersham, Buckinghamshire, UK). Purified templates underwent Sanger sequencing using standard BigDye terminator v3.1 (Applied Biosystems Inc., Foster City, USA) technology. DNA sequence chromatograms were analyzed and aligned using Sequencher 5.1 (Gene Codes corp., Ann Arbor, USA).

### 2.3 Y chromosome gene analysis

The iPLEX Sequenom MassARRAY system (Sequenom Inc., San Diego, USA) was used to genotype 29 single nucleotide polymorphisms (SNPs) from the nonrecombining Y chromosome (NRY) region as described in Sacks et al. (2013). These 29 SNPs form a panel of markers enabling differentiation between most observed dog Y chromosome haplogroups (Ardalan et al., 2012; Brown et al., 2011; Ding et al., 2011; Natanaelsson et al., 2006; Sacks et al., 2013). As in Sacks et al. (2013), we use H1 to refer to H1*, H2*, and H1 haplotypes. Five dinucleotide repeat–single tandem repeats (STR) were also genotyped from the NRY region: *650‐79.2b*,* 650‐79.3b*,* 990‐35*,* MS34CA*, and *MS41B* as previously described (Brown et al., 2011; Sacks et al., 2013).

### 2.4 Neutrality tests

To investigate whether the genetic variation present within the mitochondrial genome departs from the expectations of neutrality, Tajima's *D*, Fu and Li's F*, and Fu and Li's D* (Fu, 1997; Fu & Li, 1993; Tajima, 1989) statistics were calculated in DnaSP v 5.10.1 (Librado & Rozas, 2009). These statistics can be used to investigate the presence of demographic or selective pressures acting upon the molecular evolution of a DNA sequence. Significantly negative values indicate population expansion and/or purifying selection, whilst significantly positive values indicate balancing selection and/or a decrease in population size. Nonsignificant values indicate that the null hypothesis of neutrality cannot be rejected, that is, no indication of demographic or selective pressures. These neutrality statistics were calculated for all dingoes and then specific dingo populations separately.

### 2.5 Biogeographic analyses

Median spanning networks were calculated in Networks v4.6 (Bandelt, Forster, & Rohl, 1999; Forster et al., 2000) using the mitochondrial diagnostic region, mitochondrial control region, and Y chromosome datasets. As in Sacks et al. (2013), the median‐joining (MJ) algorithm with default settings was used (*r* = 2, ε = 0). Mitochondrial networks were created for the concatenated diagnostic region and control region separately. Control region data were analyzed separately to allow incorporation of and comparison to the existing dingo control region dataset (Oskarsson et al., 2011; Sacks et al., 2013; Savolainen et al., 2004). As the control region is not phylogenetically informative in dingoes, it was not included in the mitochondrial diagnostic region analysis (Cairns & Wilton, 2016). Mitochondrial networks are unrooted. Y chromosome networks were calculated using concatenated SNP and STR data. Y chromosome SNPs and STRs were weighted as described by Sacks et al. (2013). Briefly, STRs were weighted as: *650‐79.2b* = 5, *650‐79.3b* = 2, *990‐35 *=* *9, *MS34CA* = 6, *MS41B* = 1, and SNP loci = 90 (Brown et al., 2011; Sacks et al., 2013). Y networks were drawn using our collected data and an additional dataset including 112 Oceanic samples from Sacks et al. (2013).

To further investigate the relationship between the dingo and NGSD (Cairns & Wilton, 2016), we ran Bayesian phylogenetic analyses in Beast v1.7.5 (Drummond, Suchard, Xie, & Rambaut, 2012), allowing us to estimate the posterior probability value of the inferred relationship. Cairns and Wilton (2016) found that the posterior probability value was low (0.4), suggesting uncertainty regarding the position of the NGSD lineage within dingoes. The Bayesian analysis was conducted on a set of 124 dingoes plus 5 NGSD; three dingoes were excluded due to PCR amplification difficulties. Bayesian analyses were run in Beast v1.7.5 (Drummond, Suchard, Xie, & Rambaut, 2012) under a skyline coalescent model with a strict clock, substitution rate of 7.7027 × 10^−8^ mutations^−1^ site^−1^ year^−1^ with a standard deviation of 5.4848 × 10^−9^ (Brown & Yang, 2011; Cairns & Wilton, 2016). All runs were optimized for MCMC chain steps to ensure that the estimated sampling size of all variables was above 200 in Tracer 1.5 (Rambaut & Drummond, 2007). We sampled every 5,000 steps with a 10% burn‐in. The resulting maximum clade credibility tree was midpoint rooted.

The biogeographic distribution of each individual belonging to each mitochondrial or Y chromosome haplogroup was then plotted onto maps using the maps package (Brownrigg, Minka, Becker, & Wilks, 2014) in R, allowing visualization of the distribution of the mitochondrial and Y chromosome lineages across Australia. Simple contingency table analyses, comparing mitochondrial lineage (columns) and Y chromosome haplogroup (rows), were used to evaluate whether the distribution of Y chromosome haplogroups between the mitochondrial lineages was nonrandom.

To investigate the relationship of Y chromosome haplotypes found in dingoes and NGSD with those found in Island Southeast Asia, a network was calculated based upon data from 173 dingoes, 20 NGSD, and 79 Southeast Asian dogs, incorporating our dingo and NGSD dataset as well as the dataset from Sacks et al. (2013). The resulting network was color‐coded relative to geographical region.

### 2.6 Demographic analyses

To investigate historical patterns of demographic change in the dingo, Bayesian skyline plots were constructed in Tracer 1.5 (Rambaut & Drummond, 2007). Bayesian analyses were carried out in Beast v1.7.4 (Drummond et al., 2012) as detailed above. Skyline plots were constructed based upon the combined mitochondrial DNA dataset and each mitochondrial clade separately.

## 3 RESULTS

### 3.1 Neutrality tests

Tajima's *D* statistics were calculated for all dingoes as grouped by mitochondrial lineage using the mitochondrial diagnostic region (Table 3). Statistics could not be calculated for the NGSD as all individuals carried the same mitochondrial DNA sequence. The NW lineage statistics were found to be significantly negative, indicating the presence of purifying selection and/or population expansion. Statistics calculated for the SE lineage were negative but not significant.

**Table 3:** Nucleotide variation and neutrality statistics on mitochondrial DNA (1,706 bp) from 124 dingoes

	π	θ	Hd	Tajima's *D*	Fu and Li's F*	Fu and Li's D*
NW lineage	8.9 × 10^−4^	3.70 × 10^−3^	0.77	−2.43^a^	−4.58^a^	−4.52^a^
SE lineage	1.08 × 10^−3^	1.40 × 10^−3^	0.79	−0.65	−1.91	−2.10

a *p* < .02.

### 3.2 Biogeographic analyses

**Figure 3:**
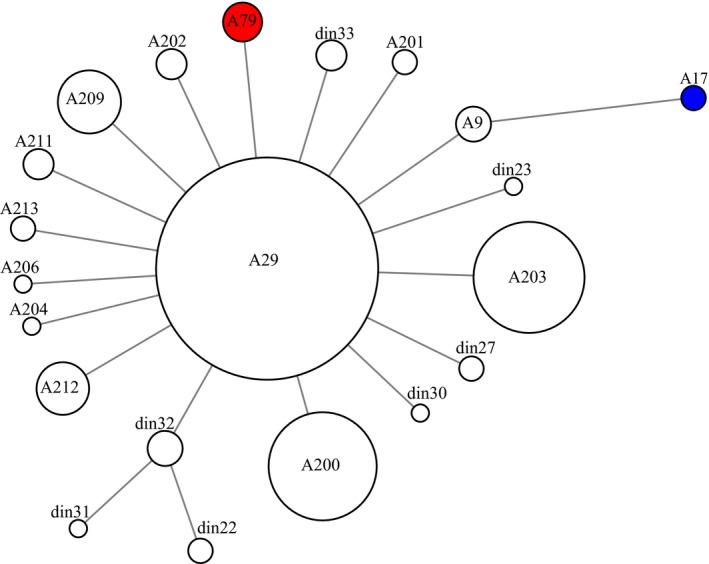
Median spanning network based on mitochondrial control region data from 450 dingoes and 23 NGSD. Red color indicates NGSD samples, whilst blue indicates a haplotype hypothesized to be introgressed from domestic dogs. Circles are proportional to the number of individuals carrying that haplotype. The network was calculated including 94 dingoes and 18 NGSD from Sacks et al. (2013) and 232 dingoes and three NGSD from Oskarsson et al. (2011)

When ignoring indels, we observed 12 mitochondrial control region (CR) haplotypes with three novel CR haplotypes in 124 dingoes (three dingoes were excluded due to PCR difficulties) and five NGSD (Table 1). The novel haplotypes (din31, din32, and din33) were found in 1–4 individuals and differed by 1–2 nucleotide substitutions from A29. One dingo carried the A9 haplotype thought to have arisen in dingoes independently from dogs (Savolainen et al., 2004). A single dingo out of 124 carried A17, a CR haplotype hypothesized to be introgressed from domestic dogs (Savolainen et al., 2004). Incorporating all the CR data from previously published studies into our own yielded a star‐shaped genetic network (Figure 3).

**Figure 4:**
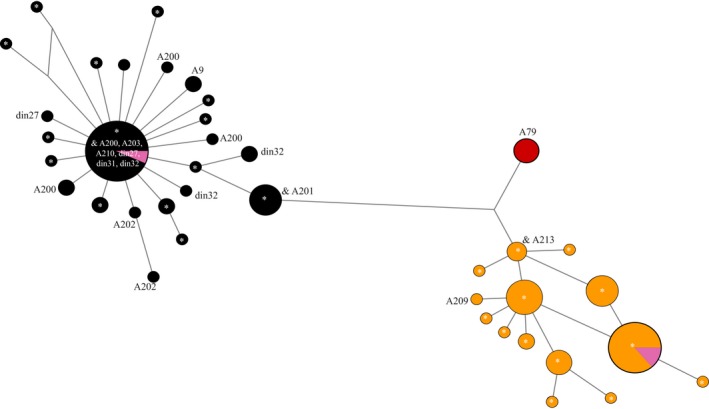
Median spanning network based upon the mitochondrial diagnostic region (1,706 bp) in 124 dingoes and five NGSD. Black coloration indicates NW lineage haplotypes, orange SE lineage haplotypes, red NGSD haplotypes, and pink captive individuals. Branch lengths are relative to the number of mutations separating mitochondrial haplotypes. Mitochondrial control region haplotypes are shown with A29 depicted as * and less common haplotypes as text

**Figure 5:**
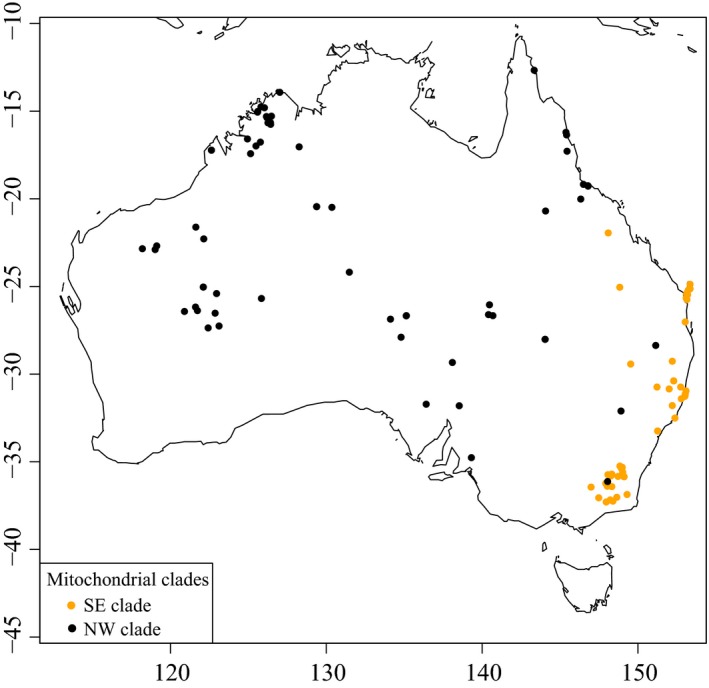
Biogeographical map of 120 dingoes and their mitochondrial lineage designation. Black circles indicate NW lineage haplotypes and orange SE lineage haplotypes. Only wild dingoes were plotted onto the map

A total of 39 mitochondrial diagnostic region haplotypes were observed in 124 dingoes and 5 NGSD (Table 1). As with the CR data, none was consistent with non‐dingo mitochondrial lineages. The 5 NGSD all carried the same haplotype. The mitochondrial diagnostic region network displays a more interesting pattern consistent with the presence of two mitochondrial haplogroups, SE and NW, in Australia (Figure 4). The genetic network also corroborated the close relationship between the SE lineage and NGSD. The biogeographic distribution of the two mitochondrial haplogroups across Australia was plotted, indicating strong geographic subdivision with limited mixing between the two populations (Figure 5). The SE mitochondrial lineage was restricted to Fraser Island and the southeastern coastal region of Australia (Queensland, New South Wales, Australian Capital Territory and Victoria), whilst the NW mitochondrial lineage was widespread from Western Australia to northern/central Queensland and south into South Australia. A single NW mitochondrial lineage individual was observed within the Australian Alpine region. Within the captive dingo population both NW and SE haplogroups were observed. Captive animals were not plotted on the map.

**Figure 6:**
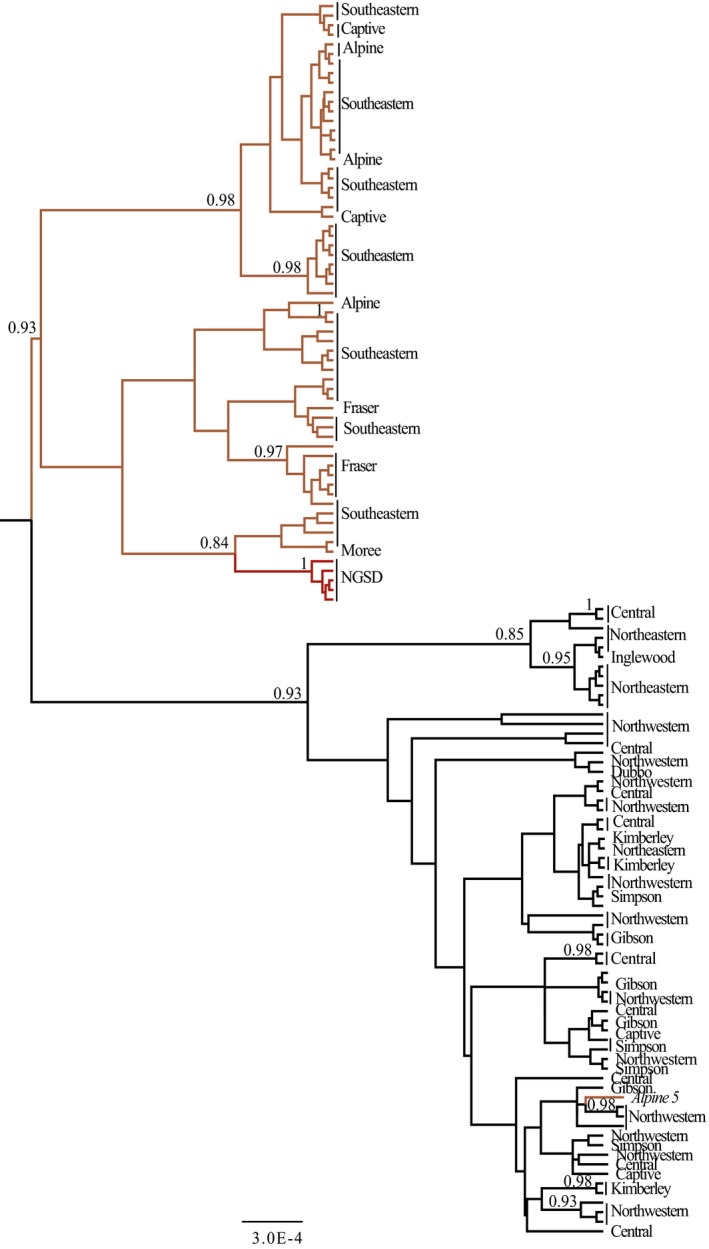
Bayesian analysis of 124 dingo and five NGSD mitochondrial diagnostic region (1,706 bp) sequences. Analyses constructed in BEAST v1.7.5 (Drummond et al., 2012) using a GTR + G + I substitution model and a constant population size coalescent model. Integers below nodes are posterior probability values and values less than 0.6 are not shown. Colors represent geographical sampling population, black for NW, orange for SE, and red for NGSD. The scale bar indicates an estimate of the average per site substitutions between two nodes

To further investigate the relationship between the dingo and NGSD, a Bayesian analysis was conducted on the combined sample of 129 animals. This included 124 dingoes and five NGSD (Table 1). This analysis corroborated the whole mitochondrial genome Bayesian phylogenetic analyses suggesting that the NGSD is more closely related to the SE dingo lineage than the NW lineage (Cairns & Wilton, 2016), with an increased posterior probability node support of 0.84 (Figure 6).

**Figure 7:**
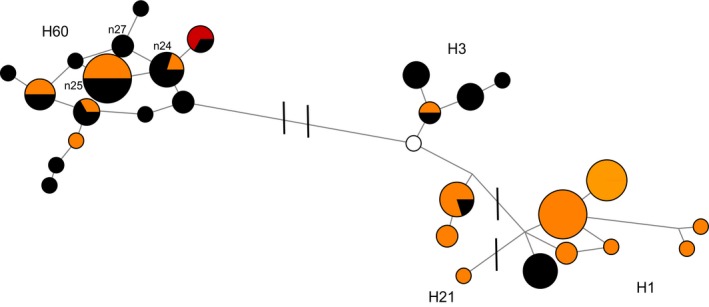
Median spanning network based upon Y chromosome SNP and STR haplotypes for 79 dingoes and two NGSD. Black coloration indicates NW mitochondrial lineage individuals, orange SE mitochondrial lineage individuals, red NGSD individuals, and white unknown mitochondrial lineage. Strokes across branches indicate the presence of Y chromosome SNP mutations differentiating between Y chromosome haplogroups. Branch lengths are relative to the number of STR mutations between Y chromosome haplotypes

**Figure 8:**
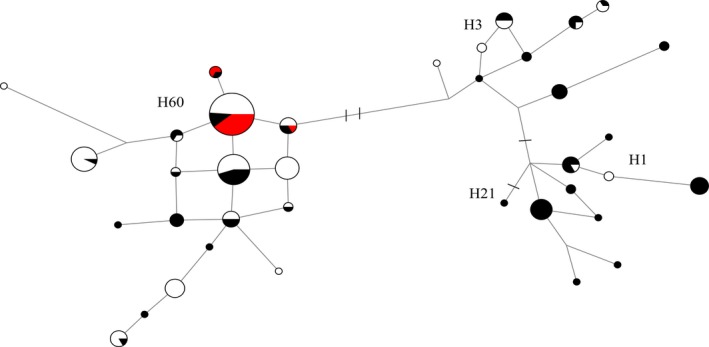
Median spanning network based upon Y chromosome SNP and STR haplotypes for 173 dingoes and 20 NGSD. Black coloration indicates dingoes from this study, red indicates NGSD individuals, and white indicates dingo samples from Sacks et al. (2013). Strokes across branches indicate the presence of Y chromosome SNP mutations differentiating between Y chromosome haplogroups. Branch lengths are relative to the number of STR mutations between Y chromosome haplotypes

We observed 30 Y chromosome haplotypes in our dataset of 79 dingoes and two NGSD (Table 1). Y chromosome network analysis identified three main haplogroups present within dingoes and NGSD, H1, H3, and H60 (Figure 7). A contingency table analysis, with two columns (mitochondrial lineage) and three rows (Y chromosome haplogroup), suggests that the distribution of Y chromosome haplogroups between the mitochondrial lineages was nonrandom in dingoes (*χ* ^2^ = 18.1, *df* = 2, *p *<* *.001). To further investigate the distribution of the Y chromosome haplogroups across Australia, we incorporated the dingo and NGSD data from Sacks et al. (2013) resulting in a total of 194 samples (Figure 8). Within the combined dataset representing 173 male dingoes and 20 NGSD, we observed an additional 6 Y chromosome SNP‐STR haplotypes (Figure 8). The 20 NGSD sampled between the two datasets all carried H60 haplotypes.

**Figure 9:**
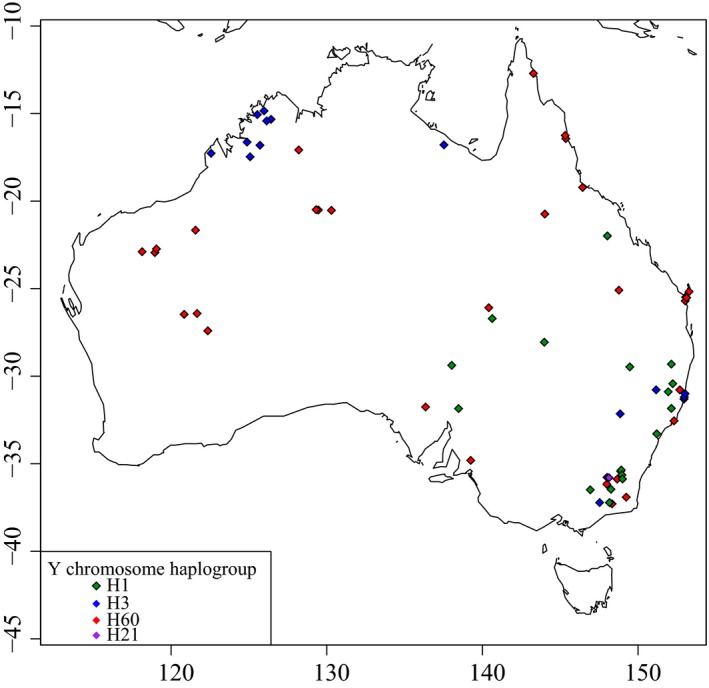
Biogeographical map of 169 dingoes and their Y chromosome haplogroup designation. Coloration indicates Y chromosome haplogroup; red for H60, blue for H3, purple for H21, and green for H1. Only wild dingoes were plotted onto the map

When Y chromosome haplogroup information was plotted on a map (Figure 9), we observed that H1 was largely restricted to the southeastern region of Australia, H3 was restricted to the southeastern and Kimberley regions, and H60 was predominantly found throughout northern, Western, and central Australia. Of the four H3 haplogroup alleles observed in the Kimberley region, all were endemic except H3_12d, which was also observed in southeastern Australia.

**Figure 10:**
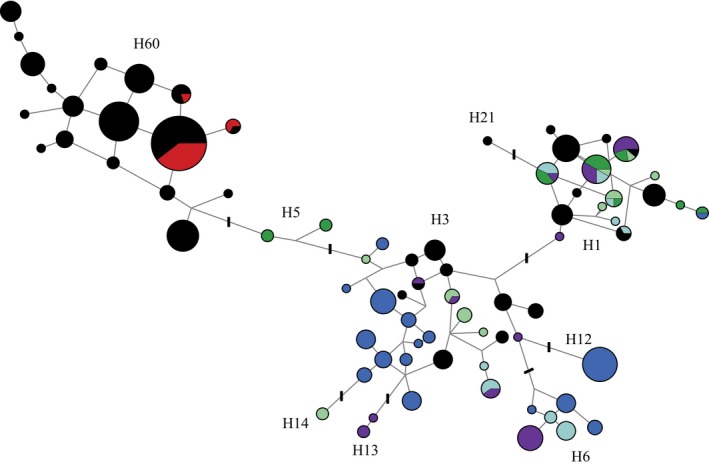
Median spanning network based upon Y chromosome SNP and STR haplotypes for 173 dingoes, 20 NGSD and 79 Southeast Asian dogs. Coloration indicates geographical region/species type: black for dingoes, red for NGSD individuals, dark green for Taiwan, purple for Thailand, light blue for Brunei, light green for Philippines, and dark blue for Bali. Strokes across branches indicate the presence of Y chromosome SNP mutations differentiating between Y chromosome haplogroups. Branch lengths are relative to the number of STR mutations between Y chromosome haplotypes

To investigate the relationship between Y chromosome haplotypes observed in dingoes and NGSD and Southeast Asian dogs, a MJ network was calculated based upon a combined dataset incorporating a total of 272 samples. This comprised 173 dingoes, 79 from our dataset and 94 from Sacks et al. (2013); 20 NGSD, two from our dataset and 18 from Sacks et al. (2013); and 79 Southeast Asian dogs from Sacks et al. (2013) (Figure 10). We observed that the H1 and H3 haplotypes found in dingoes were largely unique (not shared with SEA dogs). Further investigation, however, suggests that H1‐1c, H1‐6t, H1‐n7, and H1‐6q were observed in European domestic dog breeds (Brown et al., 2011; Sacks et al., 2013). Three novel H1 haplotypes were observed (H1‐k1, H1‐k7, and H1‐k5) that were unique to dingoes.

### 3.3 Demographic analyses

**Figure 11:**
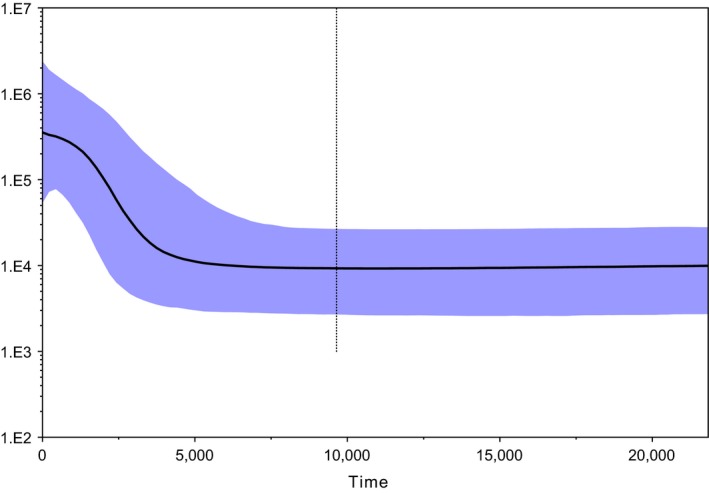
Bayesian skyline plot built using mtDNA diagnostic region (1,706 bp) sequences from 124 dingoes. Analyses constructed using a GTR + G + I substitution model and a skyline coalescent model in BEAST v1.7.5 (Drummond et al., 2012). A strict clock was enforced with a substitution rate of 7.7027 × 10^−8^ mutations^−1^ site^−1^ year^−1^ with a standard deviation of 5.4848 × 10^−9^. The skyline plot was constructed in Tracer 1.5 (Rambaut & Drummond, 2007)

**Figure 12:**
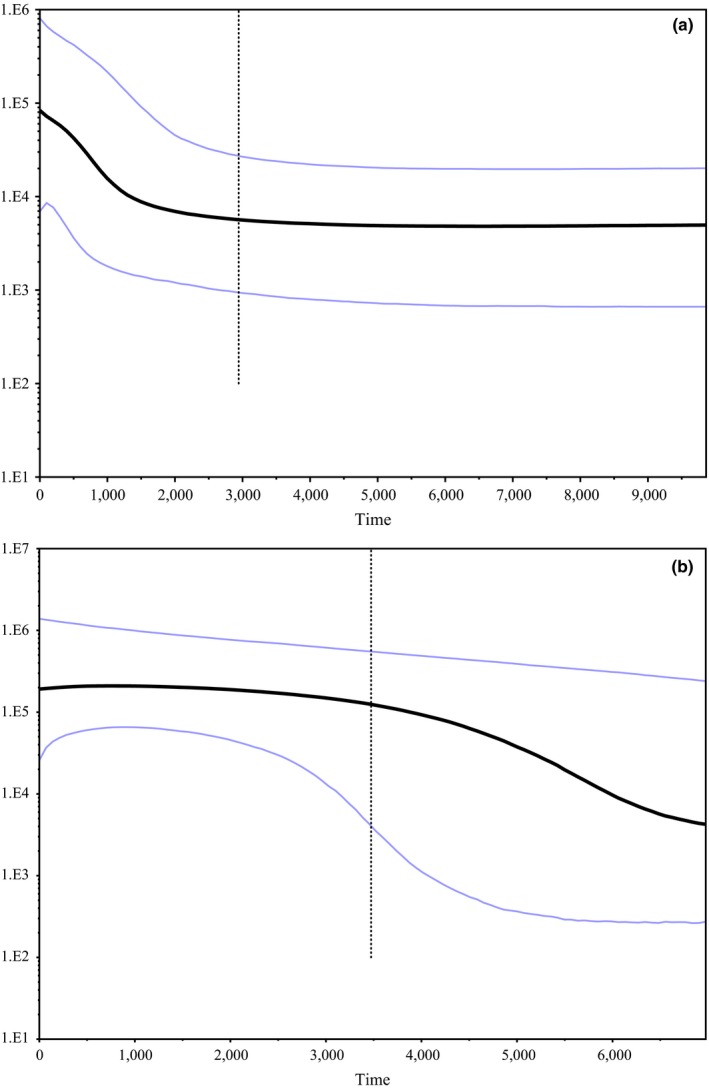
Bayesian skyline plots based on modeling on (a) 57 SE lineage or (b) 67 NW lineage dingoes using mitochondrial diagnostic region (1,706 bp) sequences. Analyses constructed using a GTR + G + I substitution model and a skyline coalescent model in BEAST v1.7.5 (Drummond et al., 2012). A strict clock was enforced with a substitution rate of 7.7027 × 10^−8^ mutations^−1^ site^−1^ year^−1^ with a standard deviation of 5.4848 × 10^−9^. The skyline plots were constructed in Tracer 1.5 (Rambaut & Drummond, 2007)

Bayesian skyline plots constructed on the combined dingo mitochondrial dataset indicate that the population was stable until approximately 5,000 years ago when it began increasing steadily (Figure 11). There was some evidence of a small decline in dingo numbers in the last 200 years. Skyline plots modeled for the individual mitochondrial clades separately suggest differences in demographic histories. The SE clade plot indicates a historically stable population size, which began increasing rapidly in the last 1,000–2,000 years (Figure 12). The NW clade plot depicts a more gradual population increase from about 6,000 years ago stabilizing approximately 3,000–4,000 years ago (Figure 12).

## 4 DISCUSSION

Understanding the ecological roles of apex predators often comes after their populations have declined to endangered levels, necessitating precautionary management (Estes et al., 2011; Ripple et al., 2014). In the case of the dingo, the findings documented here suggest the potential for sufficiently long‐standing population structure to support management for multiple locally adapted populations. Understanding the population biology, demography, and biogeography of dingoes across Australia is central to the question of how best to manage and conserve them, whilst limiting hybridization.

### 4.1 Multiple immigrations—different evolutionary lineages

Mitochondrial and Y chromosome data corroborate the presence of at least two discrete populations of dingo, NW (H60/H3), and SE (H3/H1) (Ardalan et al., 2012; Cairns & Wilton, 2016; Sacks et al., 2013). A lack of intermediate haplotypes despite broad geographical sampling suggests that the observed pattern of population structure is due to historical events. Previous studies did not observe the presence of population structure in dingoes, due to restricted sampling of the mitochondrial control region and limited geographic sampling across the Australian continent (Oskarsson et al., 2011; Sacks et al., 2013; Savolainen et al., 2004).

Our data suggest that the two divergent Y chromosome lineages observed in dingoes have different geographical origins and are plausibly the result of multiple immigrations into Australia, as postulated by Cairns and Wilton (2016). Notably, the Y haplogroups H3 and H60, which are both observed in dingoes, are not immediately related (Figure 10; Natanaelsson et al., 2006; Brown et al., 2011; Ardalan et al., 2012; Sacks et al., 2013). The H3 Y chromosome haplogroup is also observed in Southeast Asia. However, seven of the eight haplotypes observed in dingoes were endemic to Australia, indicating shared ancestry with a history of isolation between dingoes and Southeast Asian dogs (Figure 10; Brown et al., 2011; Sacks et al., 2013). On the other hand, the H60 haplogroup is unique to dingoes and NGSD and most closely related to H5, a haplogroup found in Taiwan (Figure 10; Brown et al., 2011; Ardalan et al., 2012; Sacks et al., 2013). The distribution of Y chromosome haplotypes between the two mitochondrial lineages is nonrandom, corroborating the presence of strong geographic subdivision in dingoes (Ardalan et al., 2012; Cairns & Wilton, 2016).

These data have intriguing implications for the movements of canids, and presumably humans, in Australasia. Cairns and Wilton (2016) postulate that dingoes immigrated into Australia via the now flooded land bridge between Papua New Guinea and Australia. Indeed mitochondrial data suggest that SE dingoes and the NGSD are closely related (Figures 4 and 6 and Cairns & Wilton, 2016). Y chromosome data, however, suggest that the NGSD share a closer paternal relationship with the NW lineage (Figures 7, 8, and 10; Sacks et al., 2013). Conflicting maternal and paternal histories mean the exact relationship between NGSD and the two dingo populations is uncertain. However, the close relationship does indicate that dingoes likely arrived in Australia through Papua New Guinea. Furthermore, the disparate geographical origins of the two Y chromosome haplogroups support the hypothesis that dingoes immigrated into Australia twice. A single homogeneous introduction, as suggested by Savolainen et al. (2004), is unlikely given the strong biogeographical subdivision at maternal and paternal markers and the divergent evolutionary relationships between the two populations. This suggests that in Southeast Asia and Oceania, the movements of dogs, and presumably humans, are much more complex than assumed. Indeed, genetic research finds little evidence of Neolithic or Austronesian gene flow into Australia (Bergström et al., 2016; Haak et al., 2010; van Holst Pellekaan, 2013; Karafet et al., 2005; McEvoy et al., 2010; Rasmussen et al., 2011). Intriguingly, human mitochondrial research found a pattern of continuous strong geographic subdivision dating back to approximately 50,000 years BP, after Australians first spread into the continent, with little evidence of migration between populations (Tobler et al., 2017).

### 4.2 Dating, demography, and dispersal

Demographic modeling and neutrality test results based on mitochondrial data should be treated cautiously but can give insight into modern and historical demographic patterns. Bayesian skyline plots based upon the individual mitochondrial lineages suggest that the SE population size has been stable until about 1,000 years ago, when it underwent rapid expansion (Figure 12). The NW population on the other hand has a history of gradual population expansion from approximately 4,000–6,000 years ago (Figure 12). Possibly Bayesian demographic modeling is reflective of rapid range expansion of dingoes in southeastern Australia following the decline of thylacines on the mainland, which occurred approximately 2,000 years BP (Figure 12; Johnson & Wroe, 2003; Fillios et al., 2012; Letnic et al., 2012; Prowse et al., 2014). It is also possible that the pattern of population expansion in SE dingoes is the result of extensive culling and baiting practices in southeastern Australia within the last 200 years (Fleming, Corbett, Harden, & Thomson, 2001; Wallach, Ritchie, Read, & O'Neill, 2009). The pattern of population expansion observed in the NW dingo population could be the result of long‐term but gradual range expansion after immigration into Australia. Demographic modeling on the entire dingo dataset depicts a population expansion approximately 3,000–8,000 years BP (Figure 11). Ethnographic and molecular dating suggests dingoes arrived in Australia prior to 5,000 years BP (Cairns & Wilton, 2016; Fillios & Taçon, 2016; Oskarsson et al., 2011). It should be noted that uncertainty in the demographic modeling makes it difficult to discern the approximate arrival time of dingoes or pinpoint when range expansions occurred.

Biogeographic patterns within Australia provide insight into the modern dispersal and migration of dingoes. We observed that the geographical distribution of the two mitochondrial lineages, SE and NW, exhibits strong geographical subdivision (Figure 5). Only a single instance of discordance between mitochondrial lineage and geographic origin was observed, indicating that maternal migration is limited. The geographical distribution of three Y chromosome haplogroups, H1, H3, and H60, is similar to that of the mitochondrial lineages, but more diffuse, suggesting higher levels of paternal than maternal migration (Figures 5 and 9). Introgression between the NW and SE populations seems to be west to east biased, with few H3 haplogroup individuals found in northern, Western, or central Australia. Conversely, there are some individuals in southeastern Australia harboring H60 haplogroup types, either the result of male dispersal from the NW population into the SE population or historical distribution patterns. These patterns are likely a factor of male dispersal; male dingoes and dogs range more widely and are more likely to disperse to new areas (Pal, Ghosh, & Roy, 1998; Thomson, Rose, & Kok, 1992). Human‐mediated dispersal may also be a factor in facilitating the movement of dingoes, by breaking apart pack structures through culling/baiting management practices (Corbett, 1988; Fleming et al., 2006; Glen, Dickman, Soulè, & Mackey, 2007; Thomson, 1992; Wallach, Johnson, Ritchie, & O'Neill, 2010; Wallach et al., 2009).

Contrary to demographic modeling, the neutrality test results indicate that the two dingo populations may be experiencing different demographic and/or selective pressures (Table 3 and Cairns & Wilton, 2016). These data are consistent with mitochondrial network analyses depicting a complex pattern in the SE population but a more star‐like pattern in the NW population indicative of population expansion (Figures 3 and 4). There is also evidence of a west to east biased dispersal pattern which might be the result of NW population dingoes moving into vacant niches opened up by extensive culling and baiting practices in southeastern Australia (Figure 5).

### 4.3 Introgression from modern domestic dogs

The H1 Y chromosome haplogroup is considered to be a European domestic dog haplogroup (Ardalan et al., 2012; Brown et al., 2011; Ding et al., 2011; Sacks et al., 2013), and it is often observed in domestic dog breeds or Southeast Asian dogs thought to have breed ancestry. The observation of H1 haplotypes within dingoes is suggestive of paternal introgression from European domestic dogs into dingoes (Figures 7, 8, and 10). The presence of the H1 haplogroup in genetically tested “pure” dingoes suggests that there has likely been historical, post‐European colonization, introgression from domestic dogs into dingoes. It is unlikely that the introgression is modern because the genetic test is capable of detecting hybridization events on a recent timescale (Cairns, Wilton, & Ballard, 2011; Wilton, 2001; Wilton et al., 1999). The uniparental inheritance of the Y chromosome means that a single hybridization event will be reflected in the paternal lineage of a dingo despite extensive backcrossing. The lack of non‐dingo‐like mitochondrial lineages suggests that the introgression from domestic dogs is predominately due to male domestic dogs mating with female dingoes.

The distribution of the H1 haplogroup in southeastern Australia further suggests it is likely the result of introgression (Figure 9). First, domestic dogs have been present in southeastern Australia for a longer period of time, having arrived with European colonists in 1788, allowing for a longer period of sympatry with dingoes (Corbett, 2001). Second, southeastern Australia has the densest human population, and areas with dense human populations are generally associated with higher incidences of hybridization (Stephens, 2011; Stephens et al., 2015). Thirdly, lethal management strategies such as baiting and culling are widespread in southeastern Australia due to the sheep industry (Fleming et al., 2001). Fatal management strategies are believed to lead to increased levels of hybridization due to the breakdown of pack structure (Fleming et al., 2006; Wallach et al., 2009). This finding is a significant conservation concern in the context of the genetic identity and integrity of the SE dingo population, which is under threat of extinction through introgression and ecological exclusion through lethal management programs.

### 4.4 Conservation implications

The most important implication of these data is that conservation and management efforts should be focused on maintaining the existing dingo population structure and thus treating the two populations as distinct conservation units. Care should be taken not to deliberately translocate individuals between wild populations. Captive breeding programs may need to ensure that the two dingo populations are maintained separately, with mitochondrial and Y chromosome testing used to identify population ancestry. Fraser Island dingoes appear to share NW paternal lineage ancestry but carry SE mitochondrial types; if genetic rescue is attempted for this inbred island population, then individuals from appropriate genetic lineages should be located.

Conservation groups have long described the presence of multiple morphological varieties of dingo, generally alpine, desert, and tropical; however, it is not clear whether this phenotypic variation is associated with the genetic subdivisions or phenotypic plasticity, although the boundaries do overlap. A future study of morphological and phenotypic variation as well as genetic variation may help answer this question. It is possible that the two dingo populations have different ecological or biological characteristics relevant to the conservation and management of the species or its role in specific ecosystems. Patterns of genetic subdivision in other large carnivores have been linked to ecologically relevant characteristics such as neonatal dispersal (Sacks, Bannasch, Chomel, & Ernest, 2008; Sacks, Brown, & Ernest, 2004), prey specialization (Carmichael, Nagy, Larter, & Strobeck, 2001), environmental climes (Carmichael et al., 2001; Rueness, Jorde et al., 2003; Rueness, Stenseth et al., 2003; Stenseth et al., 2004), and sociality (Randall, Pollinger, Argaw, Macdonald, & Wayne, 2010). This is a key knowledge gap, which needs to be interrogated by future ecological research.

The presence of the H1 haplogroup in southeastern Australia has important implications for conservation and future management strategies; namely, it highlights the importance of inhibiting further hybridization. Neutering male dogs and/or restricting them from reproducing with wild dingoes may help achieve this. Particularly, best practice should dictate that pet, livestock guardian, or working dogs in rural localities should be neutered or chemically castrated to avoid further risk of hybridization. Widespread lethal control measures are shown to also facilitate hybridization by breaking apart pack structures (Wallach et al., 2009). Alternative livestock protection measures should be explored, such as livestock guardians and improved dog‐proof fencing (van Bommel & Johnson, 2012; Fleming et al., 2001). This observation also suggests the need for a higher accuracy “next generation” DNA test for distinguishing dingoes from hybrids; the current method is likely sufficient for monitoring wild populations but not for captive breeding programs.

Knowledge concerning levels of genetic integrity in wild populations is necessary to inform management and conservation programs. The southeastern population of dingoes is under particular extinction pressure from both fatal management strategies and hybridization; steps should be taken to preserve this population before it is too late. A broad genetic survey of dingoes in National Parks and State Forests in southeastern states would be needed to pinpoint high dingo ancestry populations and thus where to focus conservation efforts. Currently state and federal legislation do not protect the dingo sufficiently and allow widespread fatal control measures (Davis, 2001; Downward & Bromell, 1990; Fleming et al., 2001, 2006). Revision of legislation must be achieved to reflect the ecological, cultural, and taxonomic importance of the dingo, balancing the need to conserve this enigmatic canine with any agricultural concerns.

## 5 CONCLUSIONS

Distinct populations of apex consumers can exhibit different behaviors and prey on disparate trophic niches (Paiva, Fagundes, Romão, Gouveia, & Ramos, 2016). These differences could be due to ecological plasticity or genetically inherited differences. This study corroborates the presence of at least two dingo populations in Australia. It is plausible, given the divergent evolutionary histories of these populations that they are the result of multiple immigrations into Australia via the now flooded land bridge between Papua New Guinea and Australia. The two dingo populations are geographically subdivided, with one restricted to the southeast of Australia and the other widespread across central, northern, and Western Australia. Furthermore, there are differences in male and female dispersal as evidenced by rare matrilineal migration and more diffuse patrilineal subdivision. Demographic modeling suggests that the SE population of dingoes may have expanded either in response to the extinction of thylacines on the mainland or due to widespread lethal control management in the last 1,000 years. In contrast, the NW population appears to have been gradually expanding since the dingoes’ arrival. Further research into historical demographic patterns may help inform hypotheses concerning the arrival and spread of dingoes into Australia. There is evidence of historical, post‐European colonization, paternal introgression from domestic dogs into the SE dingo population. Conservation, management, and legislative practices need to be revised to reflect the presence of two dingo populations and to limit further hybridization particularly in the SE population.

## AUTHOR CONTRIBUTIONS

KMC completed experimental design, mitochondrial DNA data collection, analysis, and writing of manuscript. BNS and SKB provided advice concerning experimental design and analysis, collected the Y chromosome data, and edited the manuscript text. JWOB provided advice and comments concerning experimental design and manuscript text.

## CONFLICT OF INTEREST

The authors declare no conflict of interests.
